# Influence of Electron Transfer Mediators in the Pd(II)-Catalyzed Oxidative Carbonylation of Aniline

**DOI:** 10.3390/molecules30092027

**Published:** 2025-05-02

**Authors:** Andrea Vavasori, Lucio Ronchin, Luca Pietrobon, Sara Bravo

**Affiliations:** Department of Molecular Science and Nanosystems, Ca’ Foscari University of Venice, Scientific Campus via Torino n° 155, 30172 Venezia, Italy; ronchin@unive.it (L.R.); luca.pietrobon@unive.it (L.P.); sara.bravo@unive.it (S.B.)

**Keywords:** oxidative carbonylation, palladium catalyst, electron transfer mediator, phenyl isocyanate, 1,3–diphenylurea

## Abstract

Currently, the most promising alternative to the use of the phosgenation reaction, for large-scale production of isocyanates, ureas, and carbamates, appears to be the Pd-catalyzed oxidative carbonylation of arylamines. During the reaction, the Pd(II) catalytic species are reduced to Pd(0) and the addition of sacrificial oxidizing agents is usually necessary to restart the catalytic cycle. Among these oxidizing agents, molecular oxygen is undoubtedly the more appealing, from an economical and green point of view, but it is not so efficient, whereas several metal salts (named cocatalysts) can be used, able to form redox couples with Pd(0) or to act as electron transfer mediators with oxygen itself. Testing several Pd(II) complexes, metal cocatalysts, and promoters, we have found that the [PdCl_2_(dppf)]/FeCl_3_/LiBr = 1/1200/200 (mol/mol) system efficiently catalyzes the carbonylation of aniline to form 1,3–diphenylurea selectively (100%) with a TOF of ca. 1177 h^−1^. On the other hand, the addition of oxygen to such a system strongly increases the aniline conversion (0.3 MPa of O_2_ increases the TOF at ca. 3930 h^−1^), but it moves the selectivity towards the phenyl isocyanate (65%, mol/mol).

## 1. Introduction

**Scheme 1:**
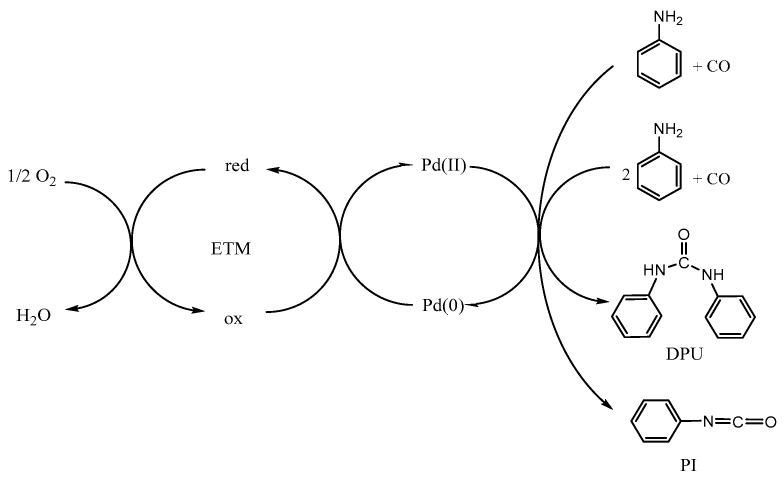
Catalytic system with electron transfer mediators (ETMs).

The replacement of hazardous and wasteful processes with more environmentally friendly and safer ones, still represents one of the most interesting challenges of current industrial and academic research [1,2,3]. Even just the use of toxic raw materials, in some cases, can make the entire process dangerous and wasteful: This is the case with phosgene, which is still considered an important building block for numerous industrial productions [4,5], but it is also a hard to handle, extremely dangerous, and toxic gas [5,6,7]. Undoubtedly, the most important examples of processes in which phosgene is still used are the large-scale production of isocyanates, ureas, carbonates, and carbamates, important raw materials for pharmaceutical, agrochemical, and polymers industries [8,9,10,11,12,13,14,15,16,17]. Nowadays, several eco-friendly alternatives to phosgenation reaction are readily available [18,19,20,21,22,23,24,25,26] and some of these have found industrial application [22,23,24,25,26]. Focusing on the production of phenyl isocyanate, the Pd-catalyzed reductive carbonylation of aryl nitro compounds [27,28,29,30,31,32,33] and the Pd-catalyzed oxidative carbonylation of aryl amines [34,35,36,37,38,39,40,41,42] proved to be the most promising routes alternative to phosgenation. For instance, from aniline, we can readily produce phenyl isocyanate and/or 1,3-diphenylurea (together with methyl N-phenylcarbamate) by using Pd(II)-based catalysts. The formation of the products, however, passes through the reduction of Pd(II) to inactive Pd(0) species and the addition of sacrificial oxidizing agents is required to complete the redox cycle (see Scheme 1) [34,35,36,37,38,39,40,41,42,43,44,45,46,47,48,49,50]. Among these oxidizing agents, the more appealing from an economical and green point of view is undoubtedly the molecular oxygen, which, however, is not so efficient with Pd(0) species (or Pd metal).

Alternatively to oxygen, several metal salts can be used able to form redox couples with Pd(0) or which can act as electron transfer mediators (ETMs) between Pd(0) and oxygen itself (see Scheme 1) [43,44,45,46,47,48,49,50].

In this regard, interesting results are reported in the literature when PdCl_2_ (or Pd(OAc)_2_) is coupled to Cu (II), Cu(I), Fe(II), Fe(III), or Ag(I) salts (named cocatalysts) [35,36,37,38,39,40,41,42,43,44,45,46,47,48,49,50]. For instance, the Pd(OAc)_2_/FeCl_2or3_/O_2_ system efficiently catalyzes the oxidative carbonylation of aniline to lead 1,3 diphenyl urea with an 88% selectivity (TOF = 1667 h^−1^ [35]). Furthermore, a promoting agent is usually added to favor such catalysis. It is plausible that it could influence the redox reaction, but it could also prevent the catalyst deactivation due to the agglomeration and the consequent precipitation of unstable Pd metal nanoparticles, which are more difficult to reoxidize [35,36,37,38,39,40,41,42,43,44,45,46,47,48,49,50].

In this paper, we tested several Pd(II)-based catalytic systems, focusing in particular on the performances of the [PdCl_2_(dppf)]/FeCl_3_/O_2_ (dppf = 1,1′-diphenylferrocene) in the presence of LiBr as the promoting agent. The best result obtained in terms of turnover frequency (TOF) was ca. 3930 h^−1^ with 65% of selectivity to phenyl isocyanate. The same system catalyzes the formation of 1,3-diphenylurea with 100% of selectivity and a TOF of ca. 1177 h^−1^, in the absence of oxygen.

## 2. Results and Discussion

### 2.1. Preliminary Experiments

A set of preliminary experiments has been carried out in the absence of oxygen using PdCl_2_ and Pd(OAc)_2_, coupled with the cocatalysts listed in Table 1. The redox couple which led to the highest catalytic activity (TOF = 40 h^−1^) was PdCl_2_/FeCl_3_, where 1,3-diphenylurea (DPU) was obtained with 85% of selectivity (at 393 K, Table 1 entry 10).

At the end of each reaction, however, palladium metal was found. Therefore, starting from the most promising data in Table 1, we decided to further investigate the PdCl_2_/FeCl_3_ system.

### 2.2. Influence of Diphosphine Ligands on the Catalytic Activity of Pd(II) Complexes

In order to improve the catalytic activity of the PdCl_2_/FeCl_3_ system, we propose to use as catalyst precursors the Pd(II)-chelating diphosphine complexes in Table 2 instead of PdCl_2_, maintaining, however, the same FeCl_3_/Pd(II) molar ratio. Such complexes were used by considering that, as is widely reported in the literature [51,52,53,54,55,56,57,58], the Pd(II)-complexes containing one P-P chelating ligand are effective in several carbonylation reactions, mainly due to the electronic characteristic of the ligand itself together with the consequent cis coordination into which the reactants are forced by the chelating effect. The latter is an important factor in a square planar configuration, characteristic of the Pd(II) complexes, as it can favor the reaction rate of the insertion reactions, involved in the catalytic cycle usually accepted for such reactions [52,53,54,55,56].

In addition, such complexes show important features and benefits from an industrial point of view as they can be readily synthesized from commercially available reagents, are stable and easily handled at room temperature, and are very soluble in common laboratory solvents (e.g., alcoholic medium).

As a matter of fact, the results reported in Table 2 show that all the complexes tested efficiently catalyze the carbonylation of aniline in the presence of FeCl_3_, leading to a higher TOF than when using PdCl_2_ (entry 1). Some of these experiments (entries 4–8) lead to 1,3-diphenylurea (DPU) with 100% of selectivity, greatly reducing or completely avoiding the deposition of Pd metal (see notes in Table 2).

**Scheme 2:**
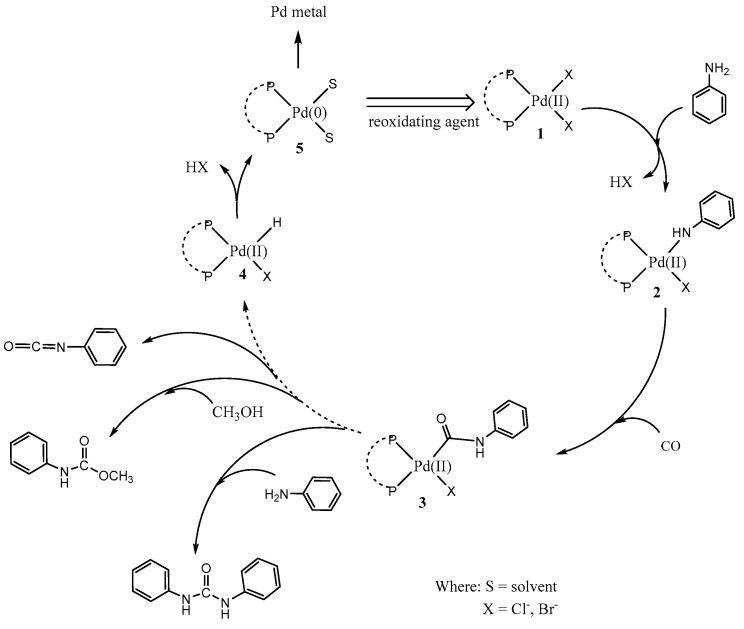
Schematization of the mainly accepted reaction mechanism for the oxidative carbonylation of aniline.

This effect was to be expected as the Pd(0) species, formed during the reaction (see species 5 in Scheme 2, [38,39,40,41,42,43]), are more stabilized against decomposition to Pd metal. Focusing on this, we have to consider that the precipitation of inactive Pd metal passes, firstly, through the formation of Pd(0) species, which in turn can decompose to Pd metal nanoparticles which are unstable towards agglomeration. The agglomeration of such nanoparticles causes their precipitation, making the reoxidation step inefficient.

Moreover, it also appears that the different electronic and steric characteristics of the chelating P–P ligands can influence TOF differently. This can be attributed to the influence of such ligands on the reactivity of all the Pd(II) and Pd(0) species formed in situ during the cycle (species 1–5 in Scheme 2).

**Scheme 3:**
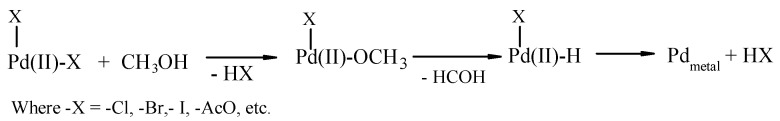
Reduction of Pd(II) to Pd metal via reaction with methanol.

In addition to this, we have to consider that, during the reaction, Pd(0) species or the Pd metal nanoparticles can form also via a parallel reaction with methanol, which is used as a solvent (see Scheme 3).

Therefore, it is plausible to suppose that by avoiding the Pd(0) decomposition and/or the Pd metal nanoparticle agglomeration, the reoxidation step can be favored and, consequently, the catalytic activity will also be increased.

According to the first hypothesis, Table 2 shows that TOF increases with the bite angle of the P-P ligands (dppm < dppe < dppp < dppb < dppf), reaching the highest value with the [PdCl_2_(dppf)] complex (at 393 K: TOF = 180 h^−1^, entry 7) [52,53,54,55]. It is widely reported that the different influence on the performance of such catalysts is related to the bite-angle of the chelating diphosphines used, due to the formation during the reaction cycle of Y–shaped Pd(0) and square planar Pd(II) species (see Scheme 2). It is known that in a square planar configuration the ideal P-P bite angle (b.a.) is 90°, while the Y–shaped compounds should be favored by P–P bite angles in the range of 94–102° [52,53]. This accords with the results in Table 2, where, by using the [PdCl_2_(PPh_3_)_2_], [PdCl_2_(dppm)] and [PdCl_2_(dppe)] complexes, only a slight increase in the TOF is obtained (entries 2–4, 60–71 h^−1^ with respect to 40 h^−1^ with PdCl_2_, entry 1), while only traces (or not at all) of Pd metal were found at the end of each reaction. This confirms that the phosphine ligands (mono-phosphines or chelating diphosphines having low bite angles) coordinated to the metal center, can stabilize the Pd(0) species in solution, avoiding the precipitation of Pd metal, but they only have a slight effect on the catalytic activity (entries 2–4).

On the other hand, P-P ligands with higher bite angles, such as dppb and dppf (98.1° and 96.1°, respectively), can stabilize both Pd(II) and Pd(0) complexes while also favoring the catalysis. This is confirmed by the increase in TOF up to 120 and 180 h^−1^, respectively, obtained without formation of Pd metal [52,53,54,55].

Moreover, by using diphosphines with larger P-Pd-P bite angles (higher than 100, Xantphos), a decrease in aniline conversion was obtained, even if the precipitation of Pd metal is still avoided (entry 8). Such larger bite-angle, which go along with decreased electron density of the metal and increased steric hindrance, makes the reductive elimination easier from the Pd(II) intermediates (species 4 in Scheme 2). Although this could favor the formation of the products, at the end of each cycle, the Pd(0) species are too stable and therefore less active towards the reoxidation, resulting in a decrease in TOF (entry 8).

In addition to the influence of the phosphine ligands, Table 2 also shows the influence of ionic ligands in the [PdX_2_(dppf)] complexes (entries 7,9,10; X = TsO = tosilate, AcO = acetate, Cl = chloride). It appears that, even if the coordinating ability of such ligands is different (TsO < AcO < Cl), they have only a slight influence on both catalytic activity and selectivity, which we have considered negligible.

Therefore, on the basis of such results, we focused on the FeCl_3_/[PdCl_2_(dppf)] system.

### 2.3. The Catalytic Activity of the FeCl_3_/[PdCl_2_(dppf)] Couple

Table 3 shows the influence of the temperature, pressure, and FeCl_3_/[PdCl_2_(dppf)] molar ratio on the catalytic activity. In all the experiments reported, only 1,3-diphenylurea (DPU) was obtained with selectivity higher than 99% (mol/mol). The TOF linearly increases by increasing the FeCl_3_ concentration (entries 1–5), rising to a plateau for Fe(III)/Pd(II) molar ratios higher than 1200/1 (637 h^−1^, entry 5). By increasing the CO pressure (entries 5–9), the TOF linearly increases up to 757 h^−1^, at 6.0 MPa (entry 5), whereas by increasing the temperature (entries 5, 10–12), it reaches a maximum value of 637 h^−1^ at 393 K (at 5.0 MPa, entry 5).

### 2.4. Influence of Promoters on the Catalytic Activity of the FeCl_3_/[PdCl_2_(dppf)] Couple

It was reported in the literature that the addition of some salts, such as TBAB (tetrabutylammonium bromide), could have a positive effect on carbonylation reactions where a Pd-based catalytic system is used [46,47,59,60,61,62]. Therefore, we have also tested the effect of some salts (named promoters) on the catalytic activity of the FeCl_3_/[PdCl_2_(dppf)] system. Given that such salts are neither a catalyst nor a cocatalyst for the carbonylation (tests in absence of Pd(II) catalyst not reported in Table 4), effectively, they make the catalytic cycle more efficient (see Table 4).

Among the promoters in Table 4, LiBr appears as the most efficient, as the TOF increases from 637 h^−1^ (without promoter, entry 1) up to 1177 h^−1^ with LiBr (entry 8). It is noteworthy that all such salts do not influence the selectivity, as in every case, only 1,3-diphenylurea was obtained (selectivity > 99%).

Regarding the LiBr/Pd(II) molar ratio, the 200/1 value was used following the results in Table 5 where the TOF reaches up to a plateau value of ca. 1177 h^−1^ when the molar ratio LiBr/Pd(II) was larger than 150/1 (see Table 5).

Furthermore, from the results reported in Table 4, we can assume that the different influence of the promoters on the TOF can be related to the nature of both the anion and the cation, since in salts having the same cation, the TOF increases in the order Cl^−^ < Br^−^ < I^−^, while in salts having the same anion, it increases in the order Na^+^ < K^+^ < TBA^+^ < Li^+^. The addition of a large excess of anions (salt/Pd = 200/1) can favor the in situ formation of more active Pd(II) species having weakly coordinating ligands (according to their coordinating ability I^−^ < Br^−^ < Cl^−^). In this case, we can also observe that such anionic ligands lead to a more pronounced effect on TOF than that observed with the ligands used in the Table 2 (entries 9 and 10). On the other hand, this influence on the TOF of the anionic ligands is superimposed on the more evident one attributable to the nature of the cation (see Table 4): Such effect could be related to a possible role played by the cations in stabilizing the Pd metal nanoparticle, according to other cases described in the literature [46,47,59,60,61,62].

In fact, it is widely reported that cations like Li^+^ and TBA^+^ (and relative counter-anions) can form layers on the surface of metal nanoparticles, generating in such a way a combination of electrostatic and steric effects, which can preserve the nanoparticles from agglomeration and from the irreversible precipitation of Pd metal power [59,60,61,62].

Supporting such hypothesis, Table 5 shows that a plateau is reached by increasing the LiBr/Pd(II) molar ratio, ascribable to the amount of promoter needed to exceed the critical micelle concentration, able to form stable micelles of Pd nanoclusters [46,47].

### 2.5. Catalytic Activity of the [PdCl_2_(dppf)]/FeCl_3_/LiBr/O_2_ Electron Transfer System

Table 6 shows the results obtained from the carbonylation of aniline carried out by using the catalytic system [PdCl_2_(dppf)]/FeCl_3_/LiBr = 1/1200/200 (mol/mol) in the presence of O_2_ as the terminal oxidizing agent. The TOF linearly increases by increasing the oxygen pressure, reaching a value of 3930 h^−1^ at partial pressure of 0.3 MPa, which is a TOF value ca. four times higher than the value reported in the literature for the system PdI_2_/FeCl_3_/O_2_ (where DPU was the main product [50]). At the same time, by increasing the oxygen partial pressure, the selectivity moves towards the formation of phenyl isocyanate (PI), reaching the value of 65% at O_2_ partial pressure of 0.3 MPa (entry 6), which is the safety limit chosen to avoid the formation of explosive gas mixtures.

Although the presence of oxygen also increases the formation of several kinds of byproducts such as N,N′–azobenzene, nitrosobenzene, etc. (this point was not explored further, <4%), the multistep electron transfer system results are very efficient. In this regard, the results in Table 6 (entry 7) also confirm that the presence of the electron transfer mediator (FeCl_3_) is necessary, as, in its absence, aniline was converted only in trace amounts.

## 3. Materials and Methods

### 3.1. Reagents

The complexes [PdX_2_(P-P)] (where –X = –Cl, –AcO; P–P = 1,1′–Bis(diphenylphosphino)ferrocene, commonly abbreviated as dppf, 1,4–Bis(diphenylphosphino)butane, commonly abbreviated as dppb, 1,3–Bis(diphenylphosphino)propane, commonly abbreviated as dppp, 1,2–Bis(diphenylphosphino)ethane, commonly abbreviated as dppe, Bis(diphenylphosphino)methane, commonly abbreviated as dppm, (9,9–Dimethyl-9H-xanthene–4,5–diyl)bis(diphenylphosphane), commonly abbreviated as Xantphos) were prepared as reported in the literature [51] and characterized by FTIR and NMR analysis. Carbon monoxide and oxygen were supplied by SIAD Company Italy (‘research grade’, purity > 99.9%). All the reagents used were Merck KGaA (Sigma-Aldrich, Darmstadt, Germany) products, not further purified.

### 3.2. Equipment and Characterization

The catalyst precursors were weighted on a Sartorious Micro balance (precision 0.001 mg).

The quantitative analyses were performed by using a high-performance liquid chromatograph (HPLC, Perkin Elmer 250) equipped with a diode array LC-235 detector and a Lichrosphere 100 (RP-18, 5 μm) column. A water acetonitrile (1:1) mixture was used as eluent and the concentrations of the reagent and products were calculated by calibration curves by using standard solutions. All the experiments were repeated three times, and the values reported in this paper are an average of these.

Although the analyses of DPU, PI, PMC, aniline, and byproduct reaction mixtures are not reliable using GC or GC-MS, due to thermal decomposition of products (in particular, DPU and PMC) at the temperature of the injector [63], we still performed some preliminary qualitative analysis to confirm the formation of the products. Such GC-MS analyses were performed on an MS Agilent apparatus 5975C Model, interfaced with an Agilent chromatograph 7890 A Model equipped with an HP50 column (30 m × 0.25 mm × 0.25 μm, oven: 318 K (3 min) to 523 K at 15°/min).

### 3.3. Experimental Setup

All the experiments were carried out in a stainless steel stirred tank reactor of ca. 100 mL of capacity. The reagents were added in a ca. 20 mL Pyrex glass beaker placed into the reactor in order to prevent contamination by metallic species due to possible corrosion of the internal surface of the reactor. In such a gas–liquid batch reactor, a magnetic stirrer and two baffles were used to ensure the absorption of the carbon monoxide into the liquid phase containing the homogeneous solution with the catalyst. The reactor was equipped with a cooling/heating jacket that together with a circulation-heating bath ensured thermal control (isothermal conditions). The temperature was monitored by using a data logger connected to a thermocouple placed inside the Pyrex glass beaker, within the liquid phase where the reaction takes place.

Carbon monoxide was supplied from a gas reservoir (a cylinder of 1 L at 15.0 MPa); in order to maintain constant pressure (5.0 MPa) during the reaction time, the reactor was connected to the gas reservoir through a pressure regulator. The pressure was monitored by using an electronic pressure sensor connected to a data logger.

Oxygen was supplied from a gas reservoir (a cylinder of 1 L at 5.0 MPa). The CO/O_2_ mixtures used in the experiments were supplied from a cylinder of 1L where we previously realized the desired partial pressures ratio by mixing CO and O_2,_ out of the explosive range. The molar ratio of the two gases was monitored by using an Agilent chromatograph 7890 A Model equipped with a molecular sieve packet column (1 m × 6.35 mm) and a thermal conductivity detector (TCD), using He as a carrier and an oven temperature of 313 K (for 3 min) increased up to 523 K at 15°/min. The cylinder containing the gas mixture was used directly to pressurize the reactor at the desired value. Moreover, the reactor was equipped with a vent valve.

### 3.4. Catalytic Reactions

The catalysis was carried out in a batch reactor of ca. 100 mL provided with a magnetic stirrer. In a typical experiment, 2.725 × 10^−3^ mmol of Pd(II) complex, together with cocatalyst/Pd(II) = 200/1 (mol/mol) and 1.50 mL (1.530 g, 16.43 mmol) of aniline, were added to 18.50 mL of solvent (methanol). The reactor was carefully flushed with CO at room temperature with stirring and then pressurized with 0.5 MPa of CO and heated up to 373 K in ca. 10 min without stirring. The pressure was then adjusted to the desired value (typically 5.0 MPa total pressure) and, while stirring, maintained at a constant throughout the experiment (1–3 h) by continuously supplying the carbon monoxide from a reservoir.

At the end of each experiment, the autoclave was quickly cooled and carefully depressurized. The reaction products were detected and quantified by the HPLC [63] (see Section 3.2).

## 4. Conclusions

The aniline carbonylation to 1,3-diphenylurea has been efficiently carried out with 100% selectivity by using the catalytic system [PdCl_2_(dppf)]/FeCl_3_/LiBr = 1/1200/200 (mol/mol), which leads to a TOF of 1177 h^−1^. Such a redox catalytic system appears to be more efficient than those reported in the literature. Moreover, with the addition of oxygen, an efficient multistep electron transfer system is realized, which leads to an increase in the TOF from 1177 h^−1^ up to ca. 3930 h^−1^, when the reaction is carried out at 393 K and 5.0 MPa of total pressure (partial pressure of O_2_ was 0.3 MPa). Under such reaction conditions, however, phenyl isocyanate is predominantly formed (a selectivity of 65% is achieved), while 1,3-diphenylurea becomes the byproduct, as the selectivity decreases to 31%. This is an interesting result, since the one-step production of phenyl isocyanate is still an important goal in this research field.

## Figures and Tables

**Table 1:** Pd(II)/metal redox couples in the oxidative carbonylation of aniline.

Entry	T	Catalyst/Cocatalyst	TOF	Selectivity		
n°	K		h^−1^	PI% ^a^	DPU% ^b^	PMC% ^c^
1	373	PdCl_2_/CuCl_2_	9	2	90	8
2	373	PdCl_2_/FeCl_3_	26	8	70	22
3	373	Pd(AcO)_2_/FeCl_3_	22	2	78	20
4	373	PdCl_2_/NiCl_2_	12	4	75	21
5	373	PdCl_2_/CeBr_3_	5	18	50	32
6	373	PdCl_2_/Mn(AcO)_2_	10	2	80	18
7	373	Pd(AcO)_2_/Co(AcO)_2_	7	8	85	7
8	373	Pd(AcO)_2_/NiCl_2_	11	n.d.	88	12
9	393	PdCl_2_/CuCl_2_	16	n.d.	95	5
10	393	PdCl_2_/FeCl_3_	40	5	85	10
11	393	Pd(AcO)_2_/FeCl_3_	38	10	80	10
12	393	PdCl_2_/NiCl_2_	23	2	86	12
13	393	PdCl_2_/CeBr_3_	8	11	63	26
14	393	PdCl_2_/Mn(AcO)_2_	12	n.d.	82	18
15	393	Pd(AcO)_2_/Co(AcO)_2_	15	3	90	7
16	393	Pd(AcO)_2_/NiCl_2_	22	n.d.	88	12

Run conditions: PdX_2_ = 2.725 × 10^−3^ mmol (X = Cl, OAc), cocatalyst/Pd(II) = 200/1 (mol/mol), CO = 5.0 MPa, aniline = 1.50 mL (1.530 g, 16.43 mmol), NaI = 81.70 mg (NaI/Pd = 200/1), CH_3_OH = 18.5 mL, time = 3 h. n.d. = not detected. ^a^ PI = phenyl isocianate, ^b^ DPU = 1,3-diphenylurea, ^c^ PMC = N-phenyl-methylcarbamate.

**Table 2:** Influence of different Pd(II)-catalyst precursors on the activity.

Entry	Pd-Catalyst	TOF		Selectivity		Notes:
n°		h^−1^	PI molar%	DPU molar%	PMC molar%	
1	PdCl_2_	40	5	85	10	Pd metal
2	[PdCl_2_(PPh_3_)_2_]	60	1	95	4	traces of Pd metal
3	[PdCl_2_(dppm)]	65	1	96	3	traces of Pd metal
4	[PdCl_2_(dppe)]	71	traces	100	n.d.	/
5	[PdCl_2_(dppp)]	90	traces	100	n.d.	/
6	[PdCl_2_(dppb)]	120	traces	100	n.d.	/
7	[PdCl_2_(dppf)]	180	traces	100	n.d.	/
8	[PdCl_2_(Xantphos)]	80	traces	100	n.d.	/
9	[Pd(OAc)_2_(dppf)]	181	traces	100	n.d.	/
10	[Pd(TsO)_2_(dppf)]	183	traces	100	n.d.	/

Run conditions: [PdCl_2_(P-P)] = 2.725 × 10^−3^ mmol (P-P = diphosphine ligands), FeCl_3_/Pd(II) = 200/1 (mol/mol), CO = 5.0 MPa, T = 393 K, aniline = 1.50 mL (1.530 g, 16.43 mmol), CH_3_OH = 18.5 mL, time = 3 h. n.d. = not detected.

**Table 3:** Influence of FeCl_3_/Pd molar ratios, temperature, and pressure on catalytic activity.

Entry	T	CO	FeCl_3_/Pd	TOF
n°	K	MPa	mol/mol	h^−1^
1	393	5.0	100	95
2	393	5.0	200	180
3	393	5.0	500	480
4	393	5.0	800	596
5	393	5.0	1200	637
6	393	2.0	1200	225
7	393	3.0	1200	350
8	393	4.0	1200	498
9	393	6.0	1200	757
10	373	5.0	1200	320
11	383	5.0	1200	455
12	415	5.0	1200	233

Run conditions: [PdCl_2_(dppf)] = 2.725 × 10^−3^ mmol, aniline = 1.50 mL (1.530 g, 16.43 mmol), CH_3_OH= 18.5 mL, time = 1 h.

**Table 4:** Influence of different promoters on the TOF.

Entry	Promoter	TOF
		h^−1^
1	/	637
2	NaI	695
3	KI	700
4	LiI	815
5	TBAI	733
6	NaBr	685
7	KBr	695
8	LiBr	1177
9	TBAB	882
10	NaCl	645
11	KCl	650
12	LiCl	760
13	TBAC	807

Run conditions: [PdCl_2_(dppf)] = 2.725 × 10^−3^ mmol, FeCl_3_/Pd(II) = 1200/1 (mol/mol), CO = 5.0 MPa, T = 393 K, aniline = 1.50 mL (1.530 g, 16.43 mmol), promoter/Pd = 200/1, CH_3_OH = 18.5 mL, time = 1 h. TBAI = tetrabutylammonium iodide, TBAB = tetrabutylammonium bromide, TBAC = tetrabutylammonium chloride.

**Table 5:** Influence of the LiBr/[PdCl_2_(dppf)] molar ratio on the TOF.

Entry	LiBr/[PdCl_2_(dppf)]	TOF
		h^−1^
1	/	637
2	50/1	715
3	100/1	950
4	150/1	1165
5	200/1	1177
6	300/1	1170
7	500/1	1175

Run conditions: [PdCl_2_(dppf)] = 2.725 × 10^−3^ mmol, FeCl_3_/Pd(II) = 1200/1 (mol/mol), CO = 5.0 MPa, T = 393 K, aniline = 1.50 mL (1.530 g, 16.43 mmol), CH_3_OH = 18.5 mL, time = 1 h. Note.: selectivity to DPU was in all cases higher than 90% (mol/mol).

**Table 6:** Influence of oxygen partial pressure on the TOF and the selectivity.

Entry	O_2_	TOF	DPU	PI	Byproducts
n°	MPa	h^−1^	mol%	mol%	mol%
1	0	1177	100	0	n.d.
2	0.05	1900	90	10	traces
3	0.10	2150	76	24	traces
4	0.20	3120	65	34	1
5	0.25	3500	40	58	2
6	0.30	3930	31	65	4
7 ^a^	0.30	/	n.d.	n.d.	n.d.

Run conditions: [PdCl_2_(dppf)] = 2.725 × 10^−3^ mmol, FeCl_3_/Pd(II) = 1200/1 (mol/mol), CO = 5.0 MPa, T = 393 K, aniline = 1.50 mL (1.530 g, 16.43 mmol), LiBr/Pd = 200/1, CH_3_OH = 18.5 mL, time = 1 h. ^a^ without FeCl_3._ n.d.: not detected.

## Data Availability

The original contributions presented in the study are included in the article, and further inquiries can be directed to the corresponding author.
